# Polymorphic Ventricular Tachycardia From Purkinje Network Within Deep Intertrabecular Recesses in Left Ventricular Excessive Trabeculation

**DOI:** 10.1016/j.jaccas.2025.105982

**Published:** 2025-12-03

**Authors:** Tabito Kino, Akihiko Nogami, Yuichi Hanaki, Yuki Komatsu, Kazuhiko Yumoto

**Affiliations:** a Department of Cardiology, Yokohama Rosai Hospital, Yokohama, Japan; b Department of Cardiology, Institute of Medicine, University of Tsukuba, Tsukuba, Japan; c Department of Cardiology, Tokyo Heart Rhythm Hospital, Tokyo, Japan

**Keywords:** left ventricular excessive trabeculation, noncompaction, polymorphic ventricular tachycardia, Purkinje network, short-coupled ventricular premature contractions

## Abstract

**Background:**

Left ventricular excessive trabeculation (LVET) is associated with malignant ventricular arrhythmias. Polymorphic ventricular tachycardia (PVT) remains particularly challenging, with limited evidence supporting catheter ablation (CA).

**Case Summary:**

A 53-year-old woman with LVET presented with drug-refractory PVT triggered by short-coupling ventricular premature contractions. Electroanatomical mapping revealed Purkinje and delayed potentials within the left ventricular septum, localized at the base of a recessed trabecular region. CA targeting these signals successfully eliminated inducible PVT. The patient has remained free from recurrent malignant arrhythmias over 11 years of follow-up.

**Discussion:**

This case highlights the role of the Purkinje fiber network within deep trabeculations in triggering life-threatening arrhythmias. Few reports describe effective CA of PVT in LVET, underscoring the novelty and clinical relevance of this finding.

**Take-Home Message:**

Deeply recessed Purkinje fibers within LVET can serve as critical triggers for malignant ventricular arrhythmias.

## Visual Summary

**Figure:**
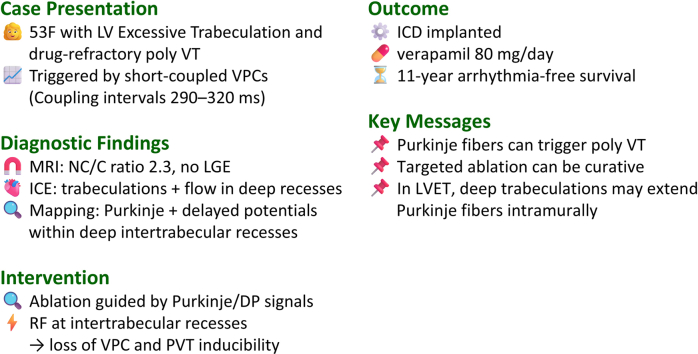

**Visual Summary:**
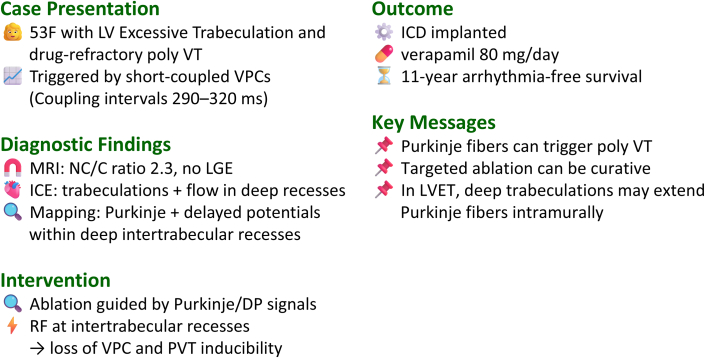
Purkinje Ablation in Left Ventricular Excessive Trabeculation

Left ventricular excessive trabeculation (LVET), often referred to as “LV noncompaction,” is a cardiomyopathy characterized by prominent trabeculations and deep intertrabecular recesses within the LV myocardium. It is thought to result from an arrest of the normal compaction process during embryonic development.^1^ Clinically, LVET is associated with a spectrum of manifestations, including heart failure, thromboembolic events, and malignant ventricular arrhythmias, all of which may significantly impact prognosis.

**Take-Home Messages**

• Deeply recessed Purkinje fibers within left ventricular excessive trabeculation can serve as critical triggers for malignant ventricular arrhythmias, as demonstrated in this case.
• An integrative approach combining imaging and electroanatomic mapping may facilitate effective catheter ablation, providing durable control in selected patients while reducing reliance on long-term pharmacological or device therapy.

Ventricular arrhythmias are frequently observed in patients with LVET, and both monomorphic and polymorphic ventricular tachycardias (PVT) have been reported.^2^ PVT, in particular, poses a serious threat due to its potential to degenerate into ventricular fibrillation (VF). The management of PVT in the context of LVET remains challenging, especially in cases refractory to pharmacological therapy. Catheter ablation (CA) has emerged as a promising therapeutic option for patients with drug-refractory ventricular arrhythmias; however, evidence supporting its efficacy in PVT-associated LVET remains limited.^3^

In this report, we present a rare case of LVET complicated by drug-refractory PVT that was successfully treated with CA of the Purkinje network within the deep intertrabecular recesses. This case underscores the feasibility and clinical relevance of ablation therapy for managing malignant arrhythmias in LVET.

## Case Report

A 53-year-old woman presented with a storm of PVT in the context of torsades de pointes, with an average cycle length of 190 ms, accompanied by syncope. Despite administration of antiarrhythmic drugs, including intravenous magnesium sulfate, lidocaine, and amiodarone, the arrhythmia remained refractory, and she was transferred to our institution for CA.

**Figure 1:**
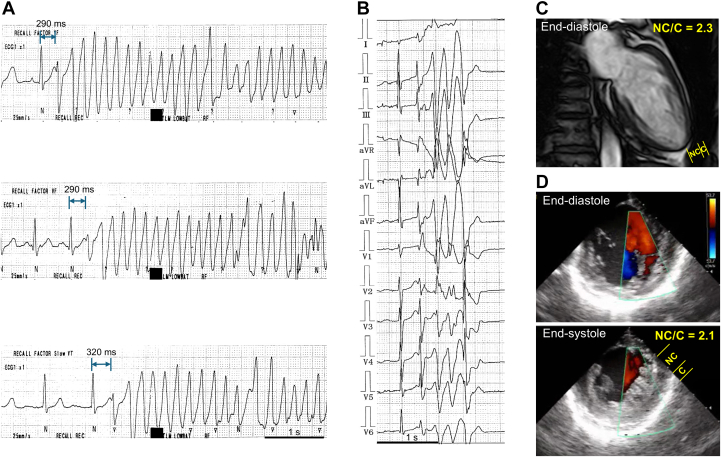
PVT Triggered by Short-Coupled VPCs and Representative Imaging Findings for the Diagnosis of LVET (A) Telemetry monitoring recording shows short-coupled VPCs (coupling interval: 290-320 ms) consistently initiating episodes of PVT. (B) A 12-lead Holter electrocardiogram detected only 173 VPCs per day, yet these VPCs frequently initiated PVT. (C) Cardiac magnetic resonance imaging at end-diastole demonstrates a noncompacted-to-compacted (NC/C) ratio of 2.3. (D) Intracardiac echocardiography (ICE) during catheter ablation demonstrated preserved LV ejection fraction, presence of false tendons, and prominent trabeculations. The NC/C ratio was 2.1, and color Doppler imaging demonstrated blood flow within deep intertrabecular recesses (see also Video 1).

She had no significant medical or family history and had never been diagnosed with cardiac abnormalities during routine health checkups. After transfer, frequent episodes of PVT persisted. Continuous telemetry revealed ventricular premature contractions (VPCs) with short coupling intervals (290-320 ms) that triggered the PVT episodes (Figure 1A). A 12-lead Holter electrocardiography detected only 173 VPCs per day; however, most of them triggered PVT, indicating high arrhythmogenicity despite the relatively low VPC burden (Figure 1B). Cardiac magnetic resonance imaging demonstrated prominent trabeculations on the endocardial side with a compact epicardial layer, absence of delayed enhancement, and a noncompacted-to-compacted ratio of 2.3, fulfilling the diagnostic criteria for LVET (Figure 1C). Intracardiac echocardiography revealed a distinct 2-layered myocardial structure. The noncompacted-to-compacted ratio was 2.1, with numerous trabeculations protruding into the LV cavity. Color Doppler imaging confirmed blood flow within the deep intertrabecular recesses (Figure 1D, Video 1).

**Figure 2:**
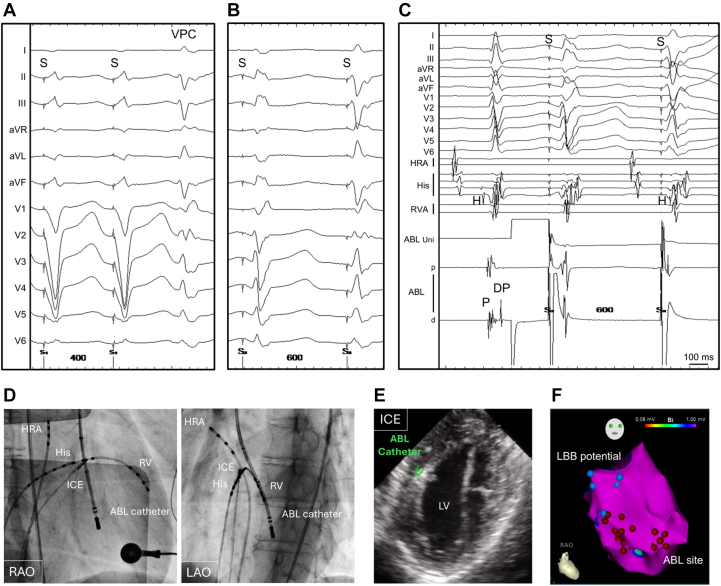
Catheter Ablation Targeting the Purkinje-Related VPCs in a Patient With LVET (A) Ventricular burst pacing induced the clinical trigger VPC. (B) Ablation (ABL) catheter was positioned at the LV septum. Pace-mapping produced 2 distinct QRS morphologies with different axes. (C) A Purkinje potential and delayed potential (DP), occurring 170 ms after the onset of the QRS complex, were recorded. Radiofrequency energy was delivered to this site. (D) Fluoroscopic images in the right anterior oblique (RAO) and left anterior oblique (LAO) views localized the site to the left posterior fascicle region of the LV. (E) Intracardiac echocardiography (ICE) confirmed that the ablation site was located at the bottom of the deep intertrabecular recesses within the noncompacted myocardial layer. The ablation catheter tip is indicated by a horseshoe-shaped green line (see also Video 2). (F) No low-voltage area was observed on the LV endocardial voltage map. Radiofrequency energy was delivered at a distal site beyond the left bundle branch (LBB) potential, where a DP was observed. HRA = high right atrium; RVA = right ventricle apex.

**Figure 3:**
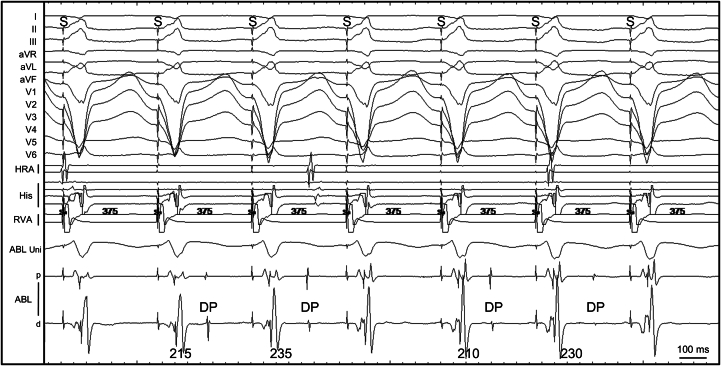
Loss of PVT Inducibility and Wenckebach-Type Block of DPs After the Nineth Ablation After 9 radiofrequency applications, neither ventricular burst pacing nor ventricular programmed stimulation induced PVT. During ventricular burst pacing, DPs exhibited a Wenckebach-type block. Abbreviations as in Figure 2.

Electroanatomical mapping of the LV was performed during sinus rhythm using CARTO 3 (Biosense Webster), but no low-voltage areas were identified. Because the trigger VPC occurred infrequently during the procedure, right ventricular burst pacing was performed and successfully induced VPCs with an electrocardiogram morphology similar to that of the spontaneous trigger VPC (Figure 2A). Based on this induced VPC, pace-mapping was conducted. When the ablation catheter was positioned at the LV septum, pacing produced 2 distinct QRS morphologies with different axes (Figure 2B). Of these, the morphology with a superior axis closely matched the spontaneous and induced VPCs. At the same site, both presystolic Purkinje potentials and delayed potentials (DPs), occurring 170 ms after QRS onset, were recorded (Figures 2C and 2D). Intracardiac echocardiography revealed that the tip of the ablation catheter (NAVISTAR, Biosense Webster) was located at the bottom of the deep intertrabecular recesses (Figure 2E, Video 2). Radiofrequency energy was delivered to the septal sites exhibiting Purkinje potentials and DPs. After 9 radiofrequency applications, VPCs were no longer inducible, and the DPs during right ventricular pacing exhibited a Wenckebach-type block (Figure 3).

**Figure 4:**
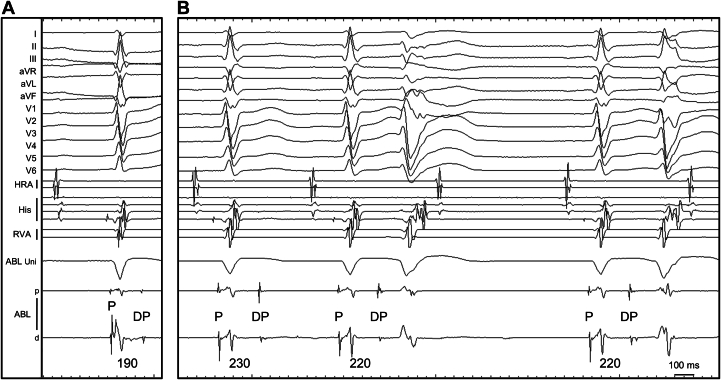
Changes in the Purkinje P–DP Interval and Induction of VPCs by Cibenzoline (A) The baseline P-DP interval was 190 ms. (B) Intravenous administration of cibenzoline (70 mg) prolonged the P–DP interval to 220 to 230 ms, and 2 types of VPCs appeared following the DPs. Even when the P–DP interval remained unchanged, 2 distinct QRS morphologies with different axes were observed, resembling the 2 pace-mapped QRS morphologies shown in Figure 2B. Abbreviations as in Figure 2.

To further evaluate the effect of ablation, a sodium channel blocker was administered. Intravenous administration of cibenzoline (70 mg) prolonged the Purkinje potential–DP (P–DP) interval from 190 to 220 or 230 ms, and 2 types of VPCs appeared following the DPs (Figure 4). Even when the coupling interval of DP remained unchanged, 2 distinct QRS morphologies with different axes were observed, resembling the 2 pace-mapped QRS morphologies shown in Figure 2B. CA guided by Purkinje potentials was then performed, targeting the DPs identified in the left posterior fascicular region (Figure 2F). The procedure was completed after confirming the noninducibility of trigger VPCs and PVT.

The patient subsequently underwent an implantation of an implantable cardioverter-defibrillator and was discharged. Thereafter, only VPCs with a longer coupling interval (350 ms) were occasionally observed. Because of poor tolerance to beta-blockers, the patient was managed with oral verapamil (80 mg/d), and no recurrence of VT or VF has been observed during an 11-year follow-up.

## Discussion

Ventricular arrhythmias in patients with LVET present unique clinical and electrophysiological challenges. In particular, PVT carries a high risk of degeneration into VF and sudden death. CA is often complicated by the anatomical complexity of the noncompacted myocardium. This case highlights several important considerations regarding arrhythmogenesis and therapeutic strategies in the context of LVET.

A notable feature of this case was the identification of short-coupled VPCs as triggers for PVT. Although the overall VPC burden was low, their marked proarrhythmic potential underscores the importance of vigilant rhythm monitoring and careful evaluation of trigger mechanisms.^4^ The presence of Purkinje potentials and DPs recorded near the left posterior fascicle suggests that the Purkinje fiber (PF) network may have played a central role in arrhythmogenesis. Previous studies have demonstrated the involvement of the Purkinje system in initiating malignant arrhythmias in both structurally normal and abnormal hearts, including those with LVET.^5^

PFs originate from embryonic ventricular cardiomyocytes that undergo lineage specification under the influence of key transcription factors such as Nkx2-5, Irx3, and Nrg1, as well as the Notch signaling pathways.^6^ Structurally, PFs are characterized by large cell diameters, high glycogen content, sparse myofibrils, and abundant expression of gap junction proteins such as Connexin 40, all of which facilitate rapid electrical conduction.^7^ Anatomically, the Purkinje system in humans is primarily located in the immediate subendocardial region of the LV, but intramural branches have also been identified within the inner half of the ventricular wall.^8^ PFs are present in the basal portion of the papillary muscles and along the crests of trabecule, forming a complex three-dimensional architecture.^9^ In LVET, excessive trabeculation and deep intramyocardial recesses may extend the Purkinje network into the mid-myocardium, potentially leading to heterogeneous patterns of depolarization and repolarization.^1^ Although CA targeting the PF network has been reported to suppress VPCs, VT, and VF, the present case is, to our knowledge, the first to demonstrate ectopic activity arising from the Purkinje system within a deeply recessed trabecular region. This finding suggests an unusual and clinically relevant deep intramyocardial extension of the Purkinje network.

LVET is recognized as a genetic cardiomyopathy. Genetic testing was performed for the patient and her family, revealing the RYR2-R2359Q (rs727504976) variant. However, its minor allele frequency is 0.00081, suggesting that it is unlikely to be a pathogenic mutation responsible for lethal arrhythmias.^10^ Notably, the patient's younger brother, who carries the same variant, exhibited neither LVET nor ventricular arrhythmias. Further investigation is planned to assess potential abnormalities in transcription factors such as Nkx2-5 and Tbx5, which are implicated in the development of the PF network during the myocardial compaction process.^6^

The current report supports a comprehensive and integrative approach to arrhythmia management in LVET, combining structural imaging, electrophysiological study, and advanced mapping. In patients with preserved systolic function and isolated electrical instability, targeted ablation therapy may offer a curative option, reducing reliance on antiarrhythmic drugs and implantable defibrillator shocks.

## Conclusions

This case illustrates the successful management of drug-refractory PVT in a patient with LVET through targeted CA of ectopic activity originating from the PF network. The findings highlight the potential role of deeply recessed PFs in arrhythmogenesis within noncompacted myocardium and underscore the value of integrating advanced imaging and electrophysiological mapping in the management of LVET-related arrhythmias. In carefully selected patients, CA may provide durable control of life-threatening ventricular arrhythmias and reduce long-term dependence on pharmacological therapy or device intervention. Further studies are warranted to establish optimal strategies for risk stratification and ablation success in this complex cardiomyopathy.

## Funding Support and Author Disclosures

The authors have reported that they have no relationships relevant to the content of this manuscript to disclose.

## Appendix

Video 1 Intracardiac Echocardiography (ICE) With Color Doppler Imaging Confirmed Blood Flow Within Deep Intertrabecular Recesses

Video 2 Intracardiac Echocardiography (ICE) Confirmed That the Ablation (ABL) Site Was Located at the Bottom of the Deep Intertrabecular Recesses Within the Noncompacted Myocardial Layer
